# *In vivo* parameter identification in arteries considering multiple levels of smooth muscle activity

**DOI:** 10.1007/s10237-021-01462-4

**Published:** 2021-05-02

**Authors:** Jan-Lucas Gade, Carl-Johan Thore, Björn Sonesson, Jonas Stålhand

**Affiliations:** 1 Department of Management and Engineering, Division of Solid Mechanics, Linköping University, Linköping, Sweden; 2 Department of Cardiothoracic and Vascular Surgery, Skåne University Hospital, Malmö, Sweden

**Keywords:** *In vivo*, Parameter identification, Artery, Smooth muscle activity

## Abstract

In this paper an existing *in vivo* parameter identification method for arteries is extended to account for smooth muscle activity. Within this method a continuum-mechanical model, whose parameters relate to the mechanical properties of the artery, is fit to clinical data by solving a minimization problem. Including smooth muscle activity in the model increases the number of parameters. This may lead to overparameterization, implying that several parameter combinations solve the minimization problem equally well and it is therefore not possible to determine which set of parameters represents the mechanical properties of the artery best. To prevent overparameterization the model is fit to clinical data measured at different levels of smooth muscle activity. Three conditions are considered for the human abdominal aorta: basal during rest; constricted, induced by lower-body negative pressure; and dilated, induced by physical exercise. By fitting the model to these three arterial conditions simultaneously a unique set of model parameters is identified and the model prediction agrees well with the clinical data.

**Supplementary Information:**

The online version contains supplementary material available at 10.1007/s10237-021-01462-4.

## Introduction

Cardiovascular diseases are the leading cause of death in the western world (Mozaffarian et al. 2016; Wilkins et al. 2017). People at risk of developing cardiovascular diseases are generally found by evaluating a risk score which comprises several biomarkers such as age, sex, blood pressure, etc. (Vlachopoulos et al. 2015; Curry et al. 2018). Risk scores are, however, not flawless, and there is a constant search for better ways to assess the risk for cardiovascular disease development. One risk marker, which has attracted a lot of attention in recent years, is arterial stiffness (Laurent et al. 2012; Van Sloten et al. 2014).

Arterial stiffness reflects the mechanical properties of the arterial wall as a whole, and its constituents and their arrangement in particular. From a mechanical point of view, elastin, collagen and smooth muscle cells are the most important wall constituents (Holzapfel et al. 2000). While elastin and collagen build up an extracellular network providing passive structural integrity to the arterial wall, the embedded smooth muscle cells modulate arterial stiffness through their ability to actively contract or relax in the short term and by synthesis of new extracellular matrix proteins in the long term (Rhodin 2014). The ability to actively change arterial stiffness within seconds is not only essential for vital blood flow circulation but also helps to counteract negative effects of cardiovascular diseases and their development. During early hypertension an increased vasoactivity stiffens the arterial wall which returns the transmural stress gradient back to its normotensive value (Fridez et al. 2002; Sehgel et al. 2013; Humphrey and Wilson 2003). With sustained hypertension the arterial wall adapts slowly by growth and remodeling and the vasoactivity returns back to baseline values (Matsumoto and Hayashi 1996; Fridez et al. 2002).

Several measures have been introduced to assess arterial stiffness in the clinic. The most prominent one is to determine the pulse wave velocity between the carotid and the femoral artery (Bramwell and Hill 1922). This noninvasive method is simple to use but averages the arterial stiffness over the aorta and assumes a constant stiffness despite the distinctive nonlinear stiffening behavior of the arterial wall (Roach and Burton 1957). Other popular measures such as the pressure-strain elastic modulus $E_\mathrm {p}$ (Peterson et al. 1960) and the stiffness index $\upbeta$ (Kawasaki et al. 1987) suffer similar limitations. Furthermore, all of these arterial stiffness measures are blood-pressure dependent (Zieff et al. 2019) and do not distinguish between arterial wall constituents.

To address these shortcomings several research groups have proposed methods that use continuum-based mechanical models whose parameters are related to the stiffness of the arterial wall constituents (Masson et al. 2008; Spronck et al. 2015; Smoljkić et al. 2015; Wittek et al. 2016; Heusinkveld et al. 2018; Gade et al. 2019; Schulze-Bauer and Holzapfel 2003). The model parameters are identified by fitting the model’s response to clinical measurements which typically comprise time-resolved pressure-radius pairs and information about the cross-sectional wall area. Information about the *in situ* axial prestretch, axial force, perivascular support, the stress-free reference configuration, and the level of smooth muscle activity is missing, however. This limited amount of *in vivo* obtainable data restricts the number of model parameters which can be uniquely identified (Stålhand and Klarbring 2005; Reesink and Spronck 2019). Introducing too many model parameters leads to overparameterization, meaning that a (nonlinear) parameter combination can be continuously changed without affecting the objective function value. This makes it impossible to determine which set of parameters represents the mechanical properties of the artery. To prevent overparameterization the complexity of the continuum-mechanical models is reduced and a common simplification concerning smooth muscle activity is to either neglect it (Smoljkić et al. 2015; Wittek et al. 2016; Heusinkveld et al. 2018; Gade et al. 2019; Schulze-Bauer and Holzapfel 2003) or to account for it but fix some model parameters to values reported in the literature (Spronck et al. 2015). An exception is the method proposed in Masson et al. (2008). Their model requires 14 parameters to be identified, however, making it very questionable whether a unique solution has been obtained (Spronck et al. 2015).

It was hypothesized in Reesink and Spronck (2019) that smooth muscle activity can be included without causing overparameterization if the arterial model is fit to multiple *in vivo* data sets collected at different levels of vascular tone. To the best of the author’s knowledge this idea has not been implemented yet. In this paper we therefore extend the mechanical model in Gade et al. (2019) to account for smooth muscle activity and fit the model to *in vivo* data sets collected at rest and while the artery was in a constricted and a dilated condition.

The paper is structured as follows. First the mechanical model from Gade et al. (2019) is described and extended to account for smooth muscle activity. Afterward the fitting procedure by means of solving a multi-objective minimization problem is discussed. The material used in this study is introduced next, followed by the results section. The paper is concluded with a discussion and a final conclusion.

## Mechanical model for arteries

The mechanical model in Gade et al. (2019) treats an artery as a homogeneous, incompressible, residual stress-free, thin-walled cylinder. *Ex vivo* in the absence of external loads, the artery has an inner radius $R_\mathrm {i}$, wall thickness *H*, and a length *L*. This state is taken to be the stress-free reference configuration. *In situ* the artery is stretched to a length *l*, which is taken to be constant throughout the cardiac cycle (Van Loon et al. 1977; Weizsäcker et al. 1983), and the inner radius and wall thickness are denoted $r_\mathrm {i}$ and *h*, respectively. In this deformed configuration, the artery is exposed to the blood pressure *P* and an axial force, where the latter cannot be measured *in vivo*. A consequence of incompressibility in combination with the constant length *l* is that the deformed cross-sectional area $A\!=\!2\pi r_\mathrm {i}h+\pi h^2$ is constant.

In the deformed configuration two sets of stresses are calculated for an artery: equilibrium stresses depending on the *in vivo* data and the unknown axial force; and constitutively determined stresses depending also on the model parameters to be identified.

### Equilibrium stresses

By stating global equilibrium in the deformed configuration, the arterial stress state reduces to a principal stress state with components in the circumferential, axial and radial direction, i.e., Laplace laws. The circumferential and axial stress are

1 $$\begin{aligned} \sigma _{\theta \theta }^\text {Lp}=\frac{Pr_\text {i}}{h} \end{aligned}$$

and

2 $$\begin{aligned} \sigma _{zz}^\text {Lp}=\frac{\pi r_\text {i}^2P+\bar{F}_\text {red}}{\pi h\left( 2r_\text {i}+h\right) }, \end{aligned}$$

respectively. The radial stress is assumed to be zero due to the thin-walled assumption, i.e., $\sigma _{rr}^\text {Lp}\!=\!0$. The axial force $\bar{F}_\mathrm {red}$ in Eq. (2), also referred to as the reduced axial force (Holzapfel et al. 2000), is reported to be approximately constant at the *in situ* axial prestretch in the physiological pressure range (Van Loon et al. 1977; Weizsäcker et al. 1983). The magnitude of $\bar{F}_\mathrm {red}$ is unknown and was estimated in Gade et al. (2019) since it could not be uniquely identified from a single *in vivo* data set.

### Constitutively determined stresses

In order to determine the arterial stress state constitutively the kinematic relationship is needed. Using the cylindrical base vectors $\mathbf{e} _\theta$, $\mathbf{e} _z$, and $\mathbf{e} _r$ the deformation gradient **F** describing the deformation out of the reference configuration into the deformed configuration becomes

3 $$\begin{aligned} \mathbf{F} =\lambda _\theta \mathbf{e} _\theta \otimes \mathbf{e} _\theta +\lambda _z\mathbf{e} _z\otimes \mathbf{e} _z+\lambda _r\mathbf{e} _r\otimes \mathbf{e} _r, \end{aligned}$$

where $\lambda _\theta$, $\lambda _z$, and $\lambda _r$ are the three principal stretches. The three stretches must satisfy the constraint

4 $$\begin{aligned} \det \mathbf{F} =\lambda _\theta \lambda _z\lambda _r=1 \end{aligned}$$

to comply with the assumed incompressible behavior and, therefore, only two stretches can be independently determined. The circumferential stretch is defined in the mid-wall as

5 $$\begin{aligned} \lambda _{\theta }=\frac{2r_\text {i}+h}{R_\text {i}+\displaystyle \sqrt{R_\text {i}^2+\lambda _z h \left( 2r_\text {i}+h \right) }}, \end{aligned}$$

where the axial stretch is taken as an independent variable. The radial stretch is implicitly given by Eq. (4) (Gade et al. 2019).

The behavior of the arterial wall is additively split into two parts: a passive and an active part (Hill 1938). The passive response of the arterial wall is derived from a strain energy density function ${\Psi }$ describing the (passive) interaction of fibers and cells. The active part $\varvec{\sigma }^\mathrm {act}$ is associated with the (active) contraction of smooth muscle cells. The constitutive equation in terms of the Cauchy stress tensor $\varvec{\sigma }$ thus reads

6 $$\begin{aligned} \varvec{\sigma }^\mathrm {mod}=-p\mathbf{I} +2\mathbf{F} \frac{\partial  {\Psi }}{\partial \mathbf{C} }{} \mathbf{F} ^\text {T}+\varvec{\sigma }^\mathrm {act}, \end{aligned}$$

where the superscript mod denotes model, *p* is a Lagrange multiplier arising from the incompressibility constraint in Eq. (4), $\mathbf{I}$ denotes the second-order identity tensor, and $\mathbf{C} \!=\!\mathbf{F} ^\text {T}{} \mathbf{F}$ is the right Cauchy-Green stretch tensor.

The Lagrange multiplier *p* can be calculated from the radial component in Eq. (6) by taking $\sigma _{rr}\!=\!0$, cf. Section 2.1. The specific forms of ${\Psi }$ and $\varvec{\sigma }^\mathrm {act}$ are introduced in the following sections.

#### Passive arterial response

The passive behavior of the arterial wall is modeled using the HGO strain energy density function ${\Psi }$ (Holzapfel et al. 2000). This strain energy is additively decomposed into an isotropic part ${\Psi }_{\text {iso}}$ and an anisotropic part ${\Psi }_{\text {aniso}}$. The isotropic part is associated with noncollagenous matrix material such as elastin and is taken as the classical neo-Hookean model (Treloar 1943)

7 $$\begin{aligned}  {\Psi }_{\text {iso}}=c\left( I_1-3\right) \text {,} \end{aligned}$$

where $c\!>\!0$ and $I_1\!=\!\text {tr}~\mathbf{C}$. The anisotropic part is associated with the embedded collagen fibers which are assumed to belong to one of two mechanically equivalent fiber families oriented along the referential unit vectors $\mathbf{M}$ and $\mathbf{N}$. Both fiber families are assumed to be symmetrically arranged around the circumferential direction with the pitch angle $\pm \beta$ in the reference configuration, so

8 $$\begin{aligned} \mathbf{M} =\cos \beta \ \mathbf{e} _\theta +\sin \beta \ \mathbf{e} _z,\quad \mathbf{N} =\cos \beta \ \mathbf{e} _\theta -\sin \beta \ \mathbf{e} _z\text {.} \end{aligned}$$

The strain energy of the fiber families is given by

9 $$\begin{aligned}  {\Psi }_{\text {aniso}} = \frac{k_1}{2\,k_2}\left( \mathrm {e}^{k_2\,\left( I_4-1\right) ^2} + \mathrm {e}^{k_2\,\left( I_6-1\right) ^2} - 2\right) \text {,} \end{aligned}$$

where

10 $$\begin{aligned} I_4\!=\!\mathbf{M} \cdot \mathbf{CM} ,\quad I_6\!=\!\mathbf{N} \cdot \mathbf{CN} , \end{aligned}$$

and $k_1,k_2\!>\!0$. The pseudo-invariants $I_4$ and $I_6$ are equal to the squared stretch along each fiber family, and using Eqs. (3), (8), and (10), it holds that

11 $$\begin{aligned} I_4 = I_6 = \lambda _\theta ^2\,\cos ^2\!\beta +\lambda _z^2\,\sin ^2\!\beta \text {.} \end{aligned}$$

The collagen fibers are assumed to only support tensile loads and buckle in compression (Holzapfel et al. 2000). The anisotropic contribution ${\Psi }_{\text {aniso}}$ is, therefore, omitted from ${\Psi }$ if $I_4, I_6\!<\!1$.

#### Active arterial response

The ability of an artery to actively constrict and dilate the lumen by changing the contracted state of the smooth muscle cells inside the wall gives rise to an active stress $\varvec{\sigma }^\mathrm {act}$. Smooth muscle cells are reported to be primarily oriented in the circumferential direction (Dobrin 2011; Rhodin 2014), and following Rachev and Hayashi (1999) we take

12 $$\begin{aligned} \varvec{\sigma }^\mathrm {act}=S\lambda _\theta f\mathbf{e} _\theta \otimes \mathbf{e} _\theta , \end{aligned}$$

where *S* is the generated isometric stress (per unit reference area) related to the level of smooth muscle activation, and *f* is a function accounting for the parabolic length–tension relationship of smooth muscle (Price et al. 1981; Cox 1978; Dobrin 1973) which satisfies $\max f\!=\!1$. Smooth muscle cells are reported to contract slowly, on the order of ten seconds or more until peak tension, but are able to maintain this state for long periods of time (Somlyo and Somlyo 1992; Dobrin 2011). We, therefore, consider vascular smooth muscle cells to contract at mean arterial blood pressure (MAP) and keep this state until the mechanical and/or chemical environment changes. Accordingly we evaluate the length–tension relationship at MAP and take

13 $$\begin{aligned} f=f\!\left( \lambda _\theta ^\mathrm {MAP}\right) =\exp \left[ -\frac{\left( \lambda _\theta ^\mathrm {opt}-\lambda _\theta ^\mathrm {MAP}\right) ^2}{2\omega ^2}\right] , \end{aligned}$$

where $\lambda _\theta ^\mathrm {opt}\!=\!1.4$ is the optimal stretch for maximum force generation (Rachev and Hayashi 1999) and $\omega \!=\!0.45$ controls the width of the parabola-like exponential function. The value for $\omega$ is chosen such that the length–tension relationship in Eq. (13) resembles the parabola suggested in Rachev and Hayashi (1999) well in the relevant interval given by $0.8\!\le \!\lambda _\theta \!\le \!1.4$.

## Parameter identification method

Let a pressure-radius data set consisting of blood-pressure and inner-radius pairs $\left( P_{qj},r_{\mathrm {i},qj}\right)$ at $j\!=\!1,\ldots ,n$ time points for arterial condition $q\!=\!1$ (basal), 2 (constricted), 3 (dilated) be given together with information about the cross-sectional area *A*. The equilibrium and constitutively determined stresses in Sect. 2 are then defined down to, respectively, the reduced axial force $\bar{F}_{\mathrm {red}}$ and the model parameters: $R_\mathrm {i}$, $\lambda _z$, *c*, $k_1$, $k_2$, $\beta$, and $S_q$. The last parameter $S_q$ is the isometric stress for arterial condition *q*. Note that the model parameters associated with the deformation $\left( R_\mathrm {i},\lambda _z\right)$ and the passive material $\left( c,k_1,k_2,\beta \right)$ do not depend on the arterial condition.

All model parameters are identified by minimizing a normalized sum of the weighted least-squares differences between the equilibrium and constitutively determined stresses for each arterial condition. The weighted least-squares difference for arterial condition *q* is defined as

14 $$ \begin{aligned}   \varepsilon _{q}  ({\kappa ,\bar{F}_{{{\text{red}}}} ,S_{q} }  )&  = \sum\limits_{{j = 1}}^{n}   \left\{w_{q} [\sigma _{{\theta \theta }}^{{{\text{mod}}}} ( {\kappa ,S_{q} ,r_{{{\text{i}},qj}} } ) \right.\\     &  - \sigma _{{\theta \theta }}^{{{\text{Lp}}}} ( {r_{{{\text{i}},qj}} ,P_{{qj}} } )]^{2}  + ( {1 - w_{q} } ) \\     & \sigma _{{zz}}^{{{\text{mod}}}} ( {\kappa ,r_{{{\text{i}},qj}} } ) - \left.\sigma _{{zz}}^{{{\text{Lp}}}} ( {r_{{{\text{i}},qj}} ,P_{{qj}} ,\bar{F}_{{{\text{red}}}} } )]^{2} \right\}  \\  \end{aligned}  $$

where $\varvec{\kappa }\!=\!\bigl (R_\text {i}\text {, }\lambda _z\text {, }c\text {, }k_1\text {, }k_2\text {, }\beta \bigl )$. The weighting factors for the three conditions are $w_1\!=\!0.5$ and $w_2\!=\!w_3\!=\!1.0$. Hence, the axial part of the individual objective $\varepsilon _q$ is only considered for the basal condition justifying the use of a single reduced axial force instead of one for each arterial condition. Furthermore, the closer a weighting factor is to zero, the more the axial part will dominate the individual objective and as a consequence the deviation of the constitutive model’s response from a constant reduced axial force with magnitude $\bar{F}_\mathrm {red}$ is penalized, see Discussion. By choosing $w_1\!=\!0.5$, an approximately constant reduced axial force is obtained for the basal condition while still achieving a high agreement in the circumferential direction.

The parameter identification for a given subject is done for the three arterial conditions simultaneously, making it a multi-objective minimization problem with the individual objectives $\varepsilon _q$. One way to handle it numerically is to minimize a weighted sum of the individual objectives, thus reducing the problem to a single-objective minimization problem. This approach is greatly affected by the magnitude of the individual objectives relative to each other and some form of normalization is typically needed. Without normalization the arterial condition with the highest objective function value dominates the parameter identification and the resulting best-fit parameters would only represent this condition well. Here we adopt a normalization scheme based on so-called Utopia and Nadir points to provide equal weight to each arterial condition (Mausser 2006).

The normalized sum of the individual objectives is

15 $$\begin{aligned} \varepsilon \!\left( \varvec{\kappa },\bar{F}_{\mathrm {red}},{{\varvec{S}}}\right) = \sum _{q=1}^3 \frac{\varepsilon _q\!\left( \varvec{\kappa },\bar{F}_{\mathrm {red}},S_q\right) -\varepsilon _q^\mathrm {Utopia}}{\varepsilon _q^\mathrm {Nadir}-\varepsilon _q^\mathrm {Utopia}}, \end{aligned}$$

where ${{\varvec{S}}}\!=\!\left( S_1,S_2,S_3\right)$ and $\varepsilon _q^\mathrm {Utopia}$ and $\varepsilon _q^\mathrm {Nadir}$ are the Utopia and Nadir point for condition *q*, respectively.

The Utopia point is the lowest least-squares difference if only arterial condition *q* is considered, i.e., the objective function value when minimizing Eq. (14). The corresponding minimization problem is, however, overparameterized, and no unique solution for the parameter vector $\left( \varvec{\kappa },\bar{F}_{\mathrm {red}},S_q\right)$ is obtained. Instead, the closely related (Utopia) minimization problem is solved: 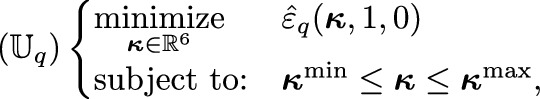
where the superscripts min and max denote lower and upper bound, respectively, and $\hat{\varepsilon }_q$ is given by Eq. (14) with $w_q$ set to 0.99. Hence, a completely passive arterial behavior, i.e., $S_q\!=\!0$, is assumed and a reduced axial force of 1 N in combination with a weighting factor of 0.99 is used for every condition to stabilize the minimization. The unique parameter vector $\varvec{\kappa }^*_q$ that minimizes $( {\mathbb{U}}_{q})$ is then used to calculate the Utopia point, i.e., $\varepsilon _q^\mathrm {Utopia}\!=\!\varepsilon _q\!( \varvec{\kappa }^*_q,1,0)$.

The Nadir point of condition *q* is the least-squares difference when the parameter vector of another Utopia point is used to evaluate $\varepsilon _q$. Hence, the Nadir point of condition $q\!=\!1$ (basal) is

16 $$\begin{aligned} \begin{aligned} \varepsilon _1^\mathrm {Nadir} = \max&\left[ \varepsilon _1\!\left( \varvec{\kappa }_2^*,1,0\right) ,\varepsilon _1\!\left( \varvec{\kappa }_3^*,1,0\right) \right] . \end{aligned} \end{aligned}$$

The Utopia and Nadir points are best-case and worst-case values, and it is easy to see that in Eq. (15), each individual objective is bounded by

17 $$\begin{aligned} 0\lesssim \frac{\varepsilon _q\!\left( \varvec{\kappa },\bar{F}_\mathrm {red},S_q\right) -\varepsilon _q^\mathrm {Utopia}}{\varepsilon _q^\mathrm {Nadir}-\varepsilon _q^\mathrm {Utopia}}\lesssim 1, \end{aligned}$$

providing equal weight to each pressure-radius loop. Note that the lower and upper bounds in Eq. (17) are only approximate due to the Utopia and Nadir point calculation, see Discussion.

The complete parameter identification problem reads: 
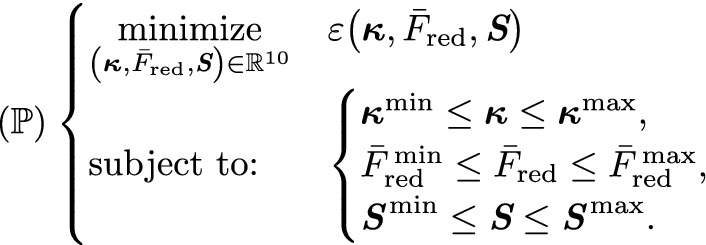
 The fitting ranges for the model parameters are motivated by experimental observations but adjusted so that they do not become active when solving $( \mathbb{P} ))$, see Table 1. The lower limit for $\lambda _z$ is set to 1.0 to prevent buckling because this phenomenon is not considered in the mechanical model.

**Table 1:** Fitting ranges for the parameter identification (Horný et al. 2011, 2014; Ferruzzi et al. 2011; Gade et al. 2019; Rachev and Hayashi 1999; Schulze-Bauer et al. 2003)

Parameter	Unit	Min	Max
$R_\text {i}$	[mm]	1	20
$\lambda _z$	[−]	1	1.5
*c*	[kPa]	0.0001	1000
$k_1$	[kPa]	0.0001	1000
$k_2$	[−]	0.0001	1000
$\beta$	[deg]	0	90
$\bar{F}_\mathrm {red}$	[N]	0	1.5
*S*	[kPa]	0	150

### Implementation

The parameter identification problem ($\mathbb{P}$) and the Utopia problems (${\mathbb{U}}_{q}$) are nonlinear and nonconvex. Such problems generally possess local solutions which are not global solutions (Nocedal and Wright 1999). We, therefore, adopt the heuristic method used in Gade et al. (2019): (i) solve ($\mathbb{P}$) and (${\mathbb{U}}_{q}$) using 100 starting points generated using Latin Hypercube sampling and take the solution with the lowest objective function value as the global solution; (ii) use the analytical gradient and Hessian of Eq. (15); (iii) and make ($\mathbb{P}$) and (${\mathbb{U}}_{q}$) more balanced by replacing *c*, $k_1$, $k_2$ and $\beta$ by scaled counterparts according to

18 $$\begin{aligned} c=\mathrm {e}^{\tilde{c}},\quad k_1=\mathrm {e}^{\tilde{k}_1},\quad k_2=\mathrm {e}^{\tilde{k}_2},\quad \beta =\arcsin \sqrt{\tilde{\beta }}. \end{aligned}$$

The minimization problems are solved in MATLAB R2019b (The MathWorks Inc., Natick, MA, USA). A *MultiStart* class is defined to solve the minimization problems from the generated starting points using the *interior-point* optimization algorithm of the function *fmincon*. The analytical gradient and Hessian of Eq. (15) are determined with Maple 2015.1 (Maplesoft, Waterloo, Ontario) and supplied to *fmincon*.

A minimal working example of the parameter identification method is found in the supplementary material.

## Material

**Fig. 1:**
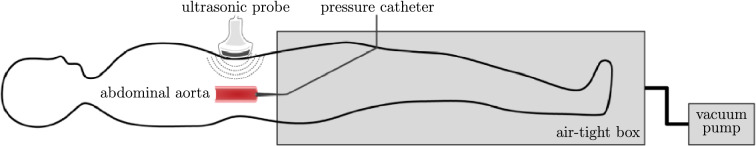
Schematic drawing of the experimental setup for simultaneous measurement of blood pressure and inner radius in the abdominal aorta during rest, lower-body negative pressure, and physical exercise

The material for this study is taken from Sonesson et al. (1997) and comes from the abdominal aorta of two healthy, non-smoking Caucasian females. Subjects I and II are 24 and 26 years of age, respectively. The blood pressure and inner radius were measured simultaneously in the supine position using a catheter (invasive) and an echo-tracking system (noninvasive), respectively. Figure 1 shows the measurement setup, and for more details about the data acquisition, the reader is referred to the original paper and Sonesson et al. (1994). Besides performing the measurements at rest, Sonesson et al. (1997) collected pressure and radius data while the aorta was in a constricted and a dilated condition^1^. The constricted condition was obtained by placing the subject’s lower body in a hermetically sealed chamber and reducing the pressure inside to near vacuum. This lower-body negative pressure technique causes pooling of blood in the lower extremities resulting in vasoconstriction (Vukasovic et al. 1990; White et al. 1996). The dilated condition was achieved by physical exercise (Green et al. 2017; MacDougall 1994) by means of asking the subject to clench their fists four times. The study was approved by the Ethics Committee at Lund University, Sweden, and all subjects gave informed consent.

**Fig. 2:**
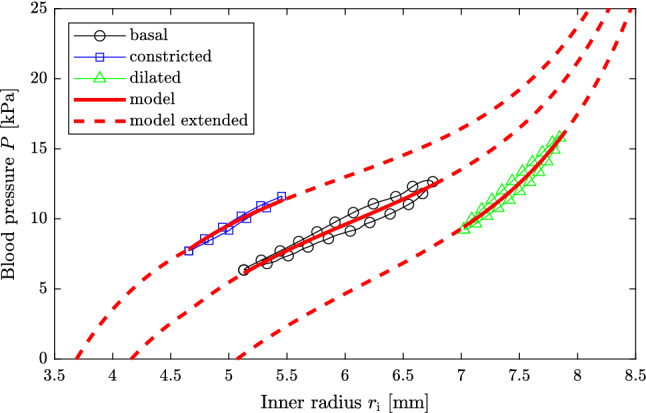
Measured pressure-radius loops and model predictions for subject I. The solid red lines are the model predictions of the three arterial conditions considered within the parameter identification. The arterial behavior outside the measured pressure-range is predicted and shown as the dashed red lines for each condition

**Fig. 3:**
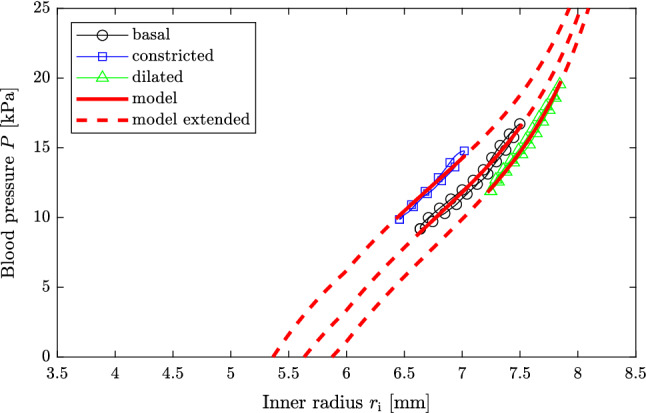
Measured pressure-radius loops and model predictions for subject II. The solid red lines are the model predictions of the three arterial conditions considered within the parameter identification. The arterial behavior outside the measured pressure-range is predicted and shown as the dashed red lines for each condition

The raw measurements are reduced to two to seven pressure-radius loops once the abdominal aorta has reached a stable state in the respective condition. From this data pressure-radius loops consisting of $n\!=\!100$ equidistant data points are created for each subject and aortic condition following Stålhand (2009). The pressure-radius loops for subjects I and II are presented in Figs. 2 and 3, respectively.

For each pressure-radius loop, MAP is calculated according to Tortora and Derrickson (2012)

19 $$\begin{aligned} P^\mathrm {MAP}=P^\mathrm {dia}+\frac{1}{3}\left( P^\mathrm {sys}-P^\mathrm {dia}\right) , \end{aligned}$$

where $P^\mathrm {dia}$ and $P^\mathrm {sys}$ are the diastolic and systolic blood pressure, respectively.

Neither the deformed wall thickness nor the deformed wall cross-sectional area were recorded in Sonesson et al. (1997). The deformed wall cross-sectional area is, therefore, estimated as $A\!=\!20.52\!+\!0.58\cdot \text {age}$, where *A* is in $\text {mm}^2$ and age is in years (Åstrand et al. 2011). The equation has been determined by evaluating the age-dependent increase of the intima-media area in the female abdominal aorta provided in Åstrand et al. (2005) and the assumption that the adventitia comprises one-third of the arterial wall (Holzapfel et al. 2007). The cross-sectional areas are accordingly estimated to $34.44~\mathrm {mm}^2$ and $35.60~\mathrm {mm}^2$ for subjects I and II, respectively.

**Table 2:** Identified parameters for subjects I and II

Parameter	Unit	Subject I	Subject II
$R_\text {i}$	[mm]	5.99	6.69
$\lambda _z$	[–]	1.32	1.15
*c*	[kPa]	22.51	54.59
$k_1$	[kPa]	25.79	52.22
$k_2$	[–]	1.72	7.50
$\beta$	[deg]	35.27	37.58
$\bar{F}_\mathrm {red}$	[N]	1.18	1.28
$S_\mathrm {basal}$	[kPa]	61.19	55.58
$S_\mathrm {constricted}$	[kPa]	136.80	93.56
$S_\mathrm {dilated}$	[kPa]	< 0.01	28.37

**Fig. 4:**
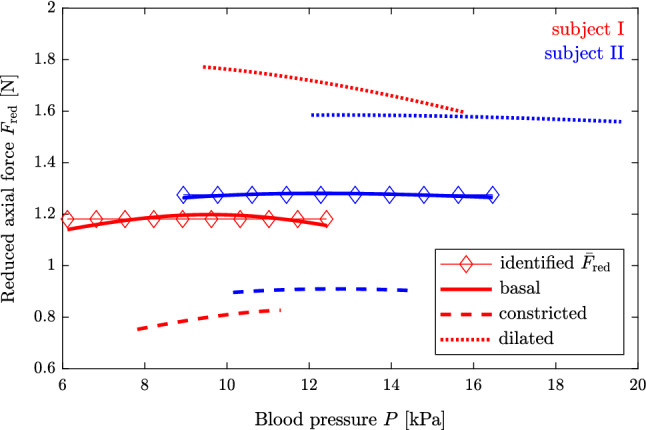
Identified reduced axial force and model prediction for both subjects. The colors red and blue are used for subjects I and II, respectively

## Results

For both subjects I and II, three pressure-radius loops measured at different levels of vascular tone are available, see Sect. 4. Hence, three levels of smooth muscle activation are included in the parameter identification and ten parameters are identified for each subject. Solving the minimization problems took less than 2 min on a hexa-core 2.9 GHz CPU and at least $70\%$ of all starting points converged to the same solution with the lowest objective function value for each subject. The identified model parameters are summarized in Table 2. Additional details about the Utopia and Nadir points are provided in the supplementary material.

In order to compare the measured pressure-radius data and the identified reduced axial force with the model prediction, the model blood pressure $P^\mathrm {mod}$ and the model reduced axial force $F_\mathrm {red}^\mathrm {mod}$ are introduced. These two quantities are calculated by replacing the Laplace stresses in Eqs. (1) and (2) with the constitutively determined stresses given by Eqs. (3), (5), (6), (7), (9), (11), (12), and (13), as

20 $$\begin{aligned} P^\mathrm {mod}=\sigma _{\theta \theta }^\mathrm {mod}\frac{h}{r_\mathrm {i}} \end{aligned}$$

and

21 $$\begin{aligned} F^\mathrm {mod}_\mathrm {red}=\sigma _{zz}^\mathrm {mod}\pi h\left( 2r_\mathrm {i}+h\right) -\pi r_\mathrm {i}^2P^\mathrm {mod}. \end{aligned}$$

**Table 3:** Agreement of the measured pressure-radius loops and the model predictions in terms of $R^2\in (-\infty ,1]$, where 1 represents a perfect fit. The column denoted ‘combined’ represents the case when the model parameters are identified considering all arterial conditions, cf. Table 2. The column ‘individual’ represents the case when the model parameters are identified using only the respective arterial condition, as when computing the Utopia point in Sect. 3

	Condition	Combined $R^2$	Individual $R^2$
Subject I	Basal	0.94	0.94
	Constricted	0.96	0.97
	Dilated	0.94	0.94
Subject II	Basal	0.97	0.98
	Constricted	0.96	0.97
	Dilated	0.98	0.98

Figures 2 and 3 show the comparison of the measured pressure-radius loops and the model predictions for subjects I and II, respectively. In order to quantify the agreement between the predicted and the measured pressure-radius loops, Table 3 displays for each arterial condition the coefficient of determination (Nash and Sutcliffe 1970)

22 $$\begin{aligned} R^2_q=1-\displaystyle \frac{\displaystyle \sum _{j=1}^n\left( P_{qj}-P^\mathrm {mod}_{qj}\right) ^2}{\displaystyle \sum _{j=1}^n\left( P_{qj}-\bar{P}_q\right) ^2}, \end{aligned}$$

where $\bar{P}_q$ is the mean of $P_{qj}$. To further put the agreement into perspective, Table 3 also contains the coefficient of determination in case the model parameters are identified for each arterial condition individually, as when computing the Utopia point in Sect. 3.

In Fig. 4, the arterial models behavior in terms of the reduced axial force is displayed for both subjects. As can be seen, the predicted reduced axial force is approximately constant in the basal condition for both subjects.

## Discussion

In this study the possibility to account for smooth muscle activity in a continuum-mechanical model is evaluated. To prevent overparameterizaton the model is fit simultaneously to three pressure-radius loops measured at different levels of smooth muscle activity. Although pressure-radius data are available in the basal, constricted and dilated condition for 19 subjects, only two young females are included in this study. The reason for this limitation is the necessity to fit the mechanical model to all three arterial conditions simultaneously. If only two conditions are considered, e.g., basal and constricted, the parameter combination can be continuously changed without affecting the objective function value, indicating that the problem is overparameterized, see supplementary material for an example. The resting and constricted arterial conditions give stable pressure-radius loops over consecutive cardiac cycles. In contrast, the physical exercise frequently caused unstable pressure-radius loops possibly due to varying smooth muscle tone or measurement errors such as involuntary motion of the ultrasonic probe. Furthermore, the pressure-radius measurements during rest and lower-body negative pressure were repeated and we only consider subjects whose repeated measurements agree^2^. Unfortunately it was only for the two included subjects that the data of all three arterial conditions contained stable pressure-radius loops over consecutive cardiac cycles and whose repeated measurements agreed with each other. In a future study it is, therefore, recommended to induce the dilated state by, e.g., pharmacological intervention (Boutouyrie et al. 2011) or the isometric handgrip exercise (Atkinson et al. 2015) to create a more stable pressure-radius response.

The proposed mechanical model in combination with the minimization scheme satisfactorily fits the measured pressure-radius loops at multiple levels of arterial tone, see Figs. 2 and 3, and the reduced axial force is reasonably constant in the basal condition, see Fig. 4. Additional figures showing the circumferential and axial stress as a function of the circumferential stretch are found in the supplementary material for both subjects.

The identified parameters for the passive part of the arterial wall are in the same range as what has been reported previously for the human abdominal aorta (Horný et al. 2011, 2014; Ferruzzi et al. 2011; Schriefl et al. 2012; Gade et al. 2019). Only the identified axial prestretch of subject II is slightly lower than what is to be expected for a 26-year-old (Horný et al. 2011). However, the deformed inner radius is quite large for subject II and the basal pressure-radius loop agrees better with the middle-aged population (Sonesson et al. 1994) which may explain the discrepancy.

To the best of the author’s knowledge, no information about the reduced axial force of the abdominal aorta in young females has been published. The identified forces have, however, a similar magnitude compared to the abdominal aorta of older subjects (Gade et al. 2019), the human thoracic aorta (Schulze-Bauer and Holzapfel 2003), and aged human iliac arteries (Schulze-Bauer et al. 2003).

The identified smooth muscle activations agree nicely with the levels suggested for basal muscular tone under normal physiological conditions, $S\!=\!50~\mathrm {kPa}$, and maximal contraction, $S\!=\!100~\mathrm {kPa}$, in case of subject II (Rachev and Hayashi 1999). For subject I, the calculated values are higher, especially under maximal contraction, cf. $S_\mathrm {constr.}\!=\!136.80~\mathrm {kPa}$ in Table 2. Despite the apparent higher smooth muscle activation for subject I, the generated active stress is instead larger for subject II, cf. Table 4. This is related to the length–tension relationship which is only $f\left( \lambda _{\theta \mathrm {,constr.}}^\mathrm {MAP}\right) \!=\!0.44$ for subject I but 0.65 for subject II. This suggests that the smooth muscle cells are at a more contraction efficient length for subject II. With respect to the dilated arterial condition, it appears that clenching the fist four times resulted in a complete relaxation of smooth muscle cells for subject I, but created a much smaller response for subject II. This behavior is expressed in Figs. 2 and 3 where the pressure-radius loop in the dilated state is shifted substantially to higher radii for subject I but barely changed for subject II.

The values of the smooth muscle activation must, however, be interpreted with caution. The active stress depends on the product *Sf*. If the length–tension relationship is changed, the identification would result in a different smooth muscle activation such that the product remains constant. It is therefore difficult to compare the identified smooth muscle activation with other studies besides Rachev and Hayashi (1999). This is particularly true if a different smooth muscle model is used (Zulliger et al. 2004; Spronck et al. 2015).

The generated active stress at MAP during the three arterial conditions is summarized in Table 4 for both subjects. To the best of the author’s knowledge this is the first study that presents *in vivo* stress values resulting from smooth muscle contraction for the human abdominal aorta. Compared to other arteries and other species, the values reported herein are in the same range (Dobrin 1978; Murtada et al. 2012; Cox et al. 1976). According to Table 4 the active stress accounts for a considerable part of the total stress in the circumferential direction already in the resting condition. This suggests that even for the elastic abdominal aorta, smooth muscle tonus is important and should be accounted for.

**Table 4:** Generated active stress $\sigma ^\mathrm {act}$ and total circumferential stress $\sigma _{\theta \theta }$ at MAP

	Condition	$\sigma ^\mathrm {act}~[\mathrm {kPa}]$	$\sigma _{\theta \theta }~[\mathrm {kPa}]$
Subject I	Basal	32.93	51.72
	Constricted	49.15	42.72
	Dilated	< 0.01	111.66
Subject II	Basal	40.66	103.98
	Constricted	60.20	94.68
	Dilated	24.60	145.06

Figures 2 and 3 unequivocally show that the human abdominal aorta can modulate its stiffness by altering smooth muscle tonus. This is especially pronounced for subject I for which not only the deformation reduces with smooth muscle contraction but also the shape of the pressure-radius loop changes substantially. While the deformation of the dilated condition is primarily governed by collagen, a shift toward the isotropic matrix is visual in the basal and constricted conditions, see Fig. 2. In order to illustrate the effect of smooth muscle contraction on arterial deformation behavior even further, the arterial model together with the identified parameters is used to predict the aortic behavior outside the physiological range for each degree of smooth muscle activation. This behavior is illustrated as the dashed red lines in Figs. 2 and 3. As one can see, the effect of smooth muscle activity is largest for low blood pressure and with increasing deformation, the three curves approach each other.

The statement that the abdominal aorta can modulate its stiffness stands in contrast to Sonesson et al. (1997) who used the same data set but concluded that the stiffness is unaffected by the sympathetic reaction due to lower-body negative pressure. Although Sonesson et al. (1997) based their conclusion on evaluating 19 subjects, compared to the two out of the 19 in this study, they assessed arterial stiffness in terms of $E_\mathrm {p}$, $\upbeta$ and pressure change-in-diameter curves. The stiffness measures $E_\mathrm {p}$ and $\upbeta$ describe the average slope of the pressure-radius relationship. As can be seen in Figs. 2 and 3 the average slope, which the model prediction represents, is almost identical for the resting and constricted condition and only differs in the dilated condition. Consequently $E_\mathrm {p}$ and $\upbeta$ are very similar in the resting and constricted condition and is only markedly higher in the dilated condition, see Table 5. Therefore, if only the resting and constricted condition are used to evaluate the effect of smooth muscle activity on arterial stiffness, no difference can be observed based on $E_\mathrm {p}$ and $\upbeta$. Similarly, if one evaluates the pressure change-in-diameter behavior no difference can be observed since the curves collapse on each other. These stiffness quantities, however, completely ignore the shift of the pressure-radius loops toward lower, in case of smooth muscle contraction, and higher radii, in case of relaxation. This highlights their inability to truly quantify arterial stiffness and the need for new alternatives, possibly based on continuum-based mechanical models such as the one used herein.

**Table 5:** Arterial stiffness in terms the pressure-strain elastic modulus $E_\mathrm {p}$ and the stiffness index $\upbeta$

	Condition	$E_\mathrm {p}~[\mathrm {kPa}]$	$\beta \left[ - \right]$
Subject I	Basal	20.21	5.86
	Constricted	22.63	7.89
	Dilated	57.11	16.16
Subject II	Basal	58.03	15.56
	Constricted	56.74	18.37
	Dilated	92.91	24.69

The choice to combine the passive mechanical model from Gade et al. (2019) with a variant of the active model from Rachev and Hayashi (1999) is based on their simplicity and the low amount of model parameters which need to be identified. Furthermore, the *in vivo* parameter identification method in Gade et al. (2019) has been (numerically) validated using a large data set.

The major difference between the passive mechanical model in Gade et al. (2019) and the ones used in other parameter identification methods (Masson et al. 2008; Spronck et al. 2015; Smoljkić et al. 2015; Heusinkveld et al. 2018) is the treatment of an artery as a thin-walled rather than a thick-walled structure. Although the geometry of an artery clearly resembles a thick-walled structure (Holzapfel et al. 2000), the existence of residual stress homogenizes the stress/stretch through the thickness (Takamizawa and Hayashi 1987; Fung 1991). This is further enhanced by smooth muscle activity (Rachev and Hayashi 1999; Humphrey and Wilson 2003) and results in an almost constant stress field throughout the arterial wall which is assumed in a thin-walled tube. Hence, the thin-walled assumption reproduces the arterial stress field to a high degree while allowing for easy analytical calculation of the gradient and Hessian of the objective function (15) which is beneficial for the parameter identification.

Another limitation is the assumption that an artery is only subjected to the blood pressure from within the lumen and prestretched in the axial direction. The *in vivo* loading situation is, however, more complex since an artery is constrained in its radial direction by surrounding tissue, organs, and bones (Humphrey 2002). This constraint is commonly incorporated by applying a perivascular pressure to the outside of an artery (Masson et al. 2008; Wittek et al. 2016). The perivascular pressure, which is reported to range from $0.67-0.93~\text {kPa}$ in a normal population (De Keulenaer et al. 2009), reduces the transmural pressure and, therefore, decreases the stress state that the arterial wall experiences. Although it is trivial to extend the used arterial model to account for perivascular pressure, because *P* in Eqs. (1) and (2) represents the transmural pressure, the outside of an artery is assumed to be traction free and as a consequence arterial stiffness is slightly overestimated. This assumption is done to avoid using population averaged data for the individual person, if it is not essential. Furthermore, perivascular pressure depends on the blood pressure (Humphrey and Na 2002) and hence differs between the three arterial conditions: it is largest in the dilated condition and smallest in the constricted condition^3^.

More advanced models to account for smooth muscle activity have been proposed and used to study the active behavior of arteries (Zulliger et al. 2004; Kroon 2009; Murtada et al. 2012; Stålhand et al. 2011; Schmitz and Böl 2011). These models consider the complete time evolution of the smooth muscle contraction, but from an *in vivo* parameter identification point of view, it is primarily the contracted state which is of importance. The active model in Rachev and Hayashi (1999) captures the intrinsic characteristics of smooth muscle contraction and requires only one additional parameter to be identified and was thus used herein.

Fitting the mechanical model to multiple pressure-radius loops simultaneously is a multi-objective minimization problem where each pressure-radius loop has its own objective. The multi-objective problem is reduced to a single-objective problem by summing up the individual objectives. There are two important aspects when solving this single-objective minimization problem: first, the functional form of the individual objectives; and second, the weighting of each individual objective.

Regarding the functional form of the individual objectives, an alternative to Eq. (14) could be:

23 $$\begin{aligned} \begin{aligned} \sum _{j=1}^n \Biggl \{&w_{q1}\Bigl [P^\text {mod}\!\left( \varvec{\kappa },S_q,r_{\text {i},qj}\right) -P_{qj}\Bigr ]^2 + \\&w_{q2} \Bigl [F_\mathrm {red}^\mathrm {mod}\!\left( \varvec{\kappa },S_q,r_{\text {i},qj}\right) -\bar{F}_\mathrm {red}\Bigr ]^2 \Biggr \}\text {,} \end{aligned} \end{aligned}$$

where $P^\mathrm {mod}$ and $F_\mathrm {red}^\mathrm {mod}$ are given by Eqs. (20) and (21). We tried using this objective, but the determination of appropriate weights $w_{11}$ and $w_{12}$ is very challenging^4^. The simplest choice is to use $w_{q2}\!=\!0$ and therefore neglect the behavior of the model in the axial direction. The problem then becomes overparameterized, however, because the parameters $\lambda _z$ and $\beta$ require some information about the axial direction. Even if a unique solution would be obtained by specifying one of those two parameters, e.g., by using population-averaged data, the behavior of the arterial model in the axial direction would still be unphysiological in terms of the magnitude of the reduced axial force and its variance throughout the cardiac cycle. The axial response of the arterial model must therefore be controlled in some way, preferably by the *in vitro* observation that the reduced axial force is approximately constant throughout the cardiac cycle (Van Loon et al. 1977; Weizsäcker et al. 1983) which we use.

The two parts in Eq. (23) do not describe analogous quantities and their relative magnitude to each other can vary substantially. Even if the first part of Eq. (23) is normalized by dividing it by $P_{qj}$ and the second part by $\bar{F}_\mathrm {red}$, the selection of appropriate weighting factors is not trivial. By converting the pressure and reduced axial force into corresponding circumferential and axial stresses using the equilibrium Eqs. (1) and (2), both parts of the individual objective represent analogous quantities and one receives an implicit normalization since both stresses have a similar magnitude. Furthermore, the choice for appropriate weighting factors appears more natural if both parts should equally contribute, cf. $w_{1}\!=\!0.5$ in Sect. 3.

In order to weigh each individual objective equally within the parameter identification problem ($\mathbb{P}$), they are normalized using their respective best-case and worst-case values, i.e., Utopia and Nadir points. In this study the Utopia and the Nadir points are calculated neglecting smooth muscle activity, i.e., only using the passive part of the mechanical model, and by using an estimate of the reduced axial force of $1~\mathrm {N}$. If the Utopia point is calculated with the complete mechanical model instead, its value is marginally smaller, at most $2\%$ for both subjects. The corresponding minimization problem is, however, overparameterized and it is not straightforward to select the argument of the Utopia point in order to calculate the Nadir point. Other possibilities such as using the argument of the Utopia point and identifying an individual smooth muscle activity and reduced axial force for the determination of the Nadir point or normalizing by simply dividing $\varepsilon _q$ by its Utopia point have been tested but the presented normalization scheme provided the best results. The well-working normalization scheme can be appreciated by examining Figures 2 and 3, and by comparing the coefficients of determination in Table 3 which barely deteriorated by fitting the model to multiple pressure-radius loops.

## Conclusion

In this study, an *in vivo* parameter identification method for arteries is extended to account for smooth muscle activity. To overcome the problem of overparameterization due to an increased number of model parameters, the continuum-mechanical model is calibrated using data measured at multiple levels of vascular tone. Despite the simplicity of the mechanical model, it fits the measured pressure-radius loops at rest, under lower-body negative pressure and during physical exercise well.

## Supplementary Information

Below is the link to the electronic supplementary material.

Supplementary file1 (PDF 386 kb)

## Footnotes

1 The measurements in the dilated condition were not published in Sonesson et al. (1997).

2 One measurement was performed during the physical exercise and another one directly afterward. Due to the dynamic nature of the exercise only the latter measurement contained stable pressure-radius loops.

3 The perivascular pressure might be close to zero or even negative during lower-body negative pressure because the pressure-radius measurements are performed in the vicinity of the pressure chamber, see Sect. 4.

4 The choice of the weighting factors for the constricted and dilated arterial condition is trivial since only the pressure part is used.
