# Supplementary material for: In vivo parameter identification in arteries considering multiple levels of smooth muscle activity
Source: Biomech Model Mechanobiol. 2021 May 2;20(4):1547–59. doi: 10.1007/s10237-021-01462-4 (PMC8298368; doi:10.1007/s10237-021-01462-4)

Supplementary material

In vivo parameter identification in arteries considering multiple levels of smooth muscle activity

Jan-Lucas Gade[1,*] Carl-Johan Thore[1] Björn Sonesson[2]
Jonas Stålhand[1]

[1]Department of Management and Engineering, Division of Solid Mechanics, Linköping University, Linköping, Sweden
[2]Department of Cardiothoracic and Vascular Surgery, Skåne University Hospital, Malmö, Sweden

[*]Corresponding author: Jan-Lucas Gade, Email: jan-lucas.gade@liu.se

Appendix

A Additional tables

A.1 Utopia and Nadir points

The identified parameters for the Utopia point of each arterial condition of subjects I and II are summarized in Tables A1 and A2, respectively. The values of the Utopia and Nadir points are also provided. Observe that while the parameters are identified with a weighting factor of $w = 0.99$, the Utopia and Nadir point are calculated with $w_1 = 0.5$ and $w_2 = w_3 = 1.0$, cf. Section 3 in the original manuscript.

Table A1 Identified parameters for the Utopia points of subject I

Parameter	Unit	Basal	Constricted	Dilated
R_i	[mm]	4.56	4.11	6.26
λ_z	[-]	1.16	1.19	1.06
c	[kPa]	25.29	15.77	87.62
k_1	[kPa]	7.74	23.08	1.41
k_2	[-]	2.62	0.0001	37.03
β	[deg]	49.75	44.10	47.72
$\bar{F}_\mathrm{red}$	[N]	1[a]	1[a]	1[a]
S	[kPa]	0[a]	0[a]	0[a]
$\varepsilon^\mathrm{Utopia}$	[kPa^2]	695	101	2536
$\varepsilon^\mathrm{Nadir}$	[kPa^2]	416960	2481800	5936001

[a]This parameter is specified.

Table A2 Identified parameters for the Utopia points of subject II

Parameter	Unit	Basal	Constricted	Dilated
R_i	[mm]	3.90	5.62	6.38
λ_z	[-]	1.50	1.08	1.05
c	[kPa]	5.41	49.11	102.60
k_1	[kPa]	3.87	31.18	14.23
k_2	[-]	0.61	5.17	20.80
β	[deg]	40.34	43.99	44.19
$\bar{F}_\mathrm{red}$	[N]	1[a]	1[a]	1[a]
S	[kPa]	0[a]	0[a]	0[a]
$\varepsilon^\mathrm{Utopia}$	[kPa^2]	566	416	926
$\varepsilon^\mathrm{Nadir}$	[kPa^2]	55830	224410	497630

[a]This parameter is specified.

A.2 Overparameterized problem

In case only two arterial conditions, e.g. basal and constricted, are used within the parameter identification, several solutions with different model parameters but the same error-function value are identified, cf. Table A3. Hence, the problem is overparameterized and no unique solution can be obtained.

Table A3 Identified parameters for subject I in case only the basal and constricted arterial condition are used in the parameter identification. The minimization problem is overparameterized and several solutions have the same objective function value

		(non-unique) Solution		
Parameter	Unit	**1**	**2**	**3**
R_i	[mm]	4.81	4.95	5.31
λ_z	[-]	1.04	1.07	1.20
c	[kPa]	24.48	25.26	28.04
k_1	[kPa]	13.56	11.77	8.31
k_2	[-]	5.82	1.18	4.23
β	[deg]	51.48	49.62	44.34
$\bar{F}_\mathrm{red}$	[N]	0.001	0.195	0.973
S_basal	[kPa]	18.92	21.90	29.25
$S_\mathrm{constricted}$	[kPa]	53.34	59.84	77.79
ε	[-]	3.35e-4	3.35e-4	3.35e-4

B Additional figures

Additional figures representing the measured pressure–radius loops and model predictions in terms of stress–stretch. Figures B1 and B2 show circumferential stress–circumferential stretch and axial stress–circumferential stretch of subject I, respectively, and Figs. B3 and B4 are analogously for subject II.

Fig. B1 Measured data and model prediction in terms of circumferential stress–circumferential stretch for subject I

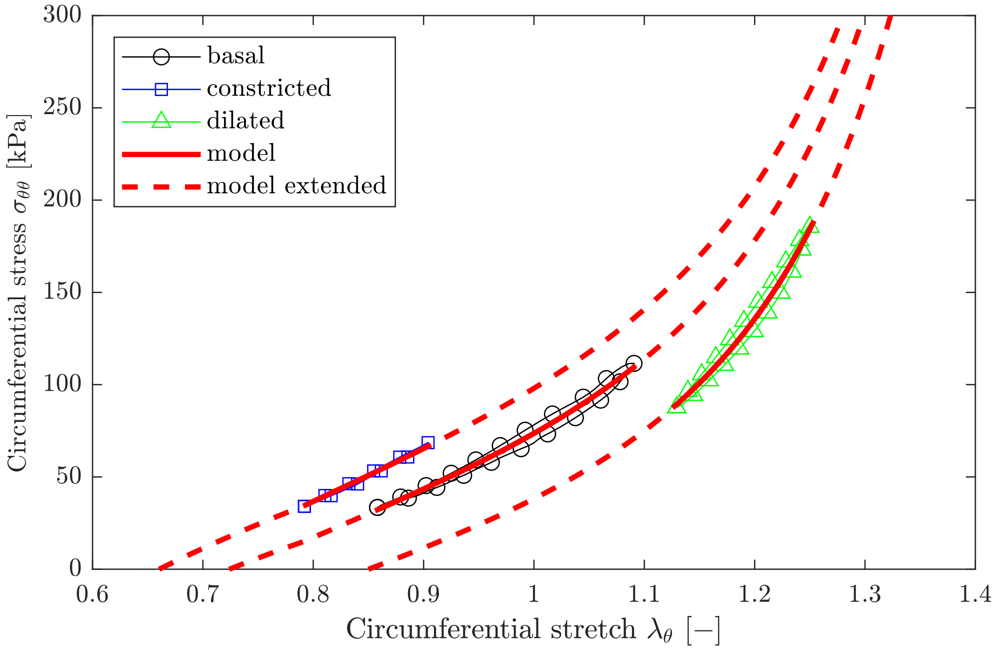

Fig. B2 Measured data and model prediction in terms of axial stress–circumferential stretch for subject I

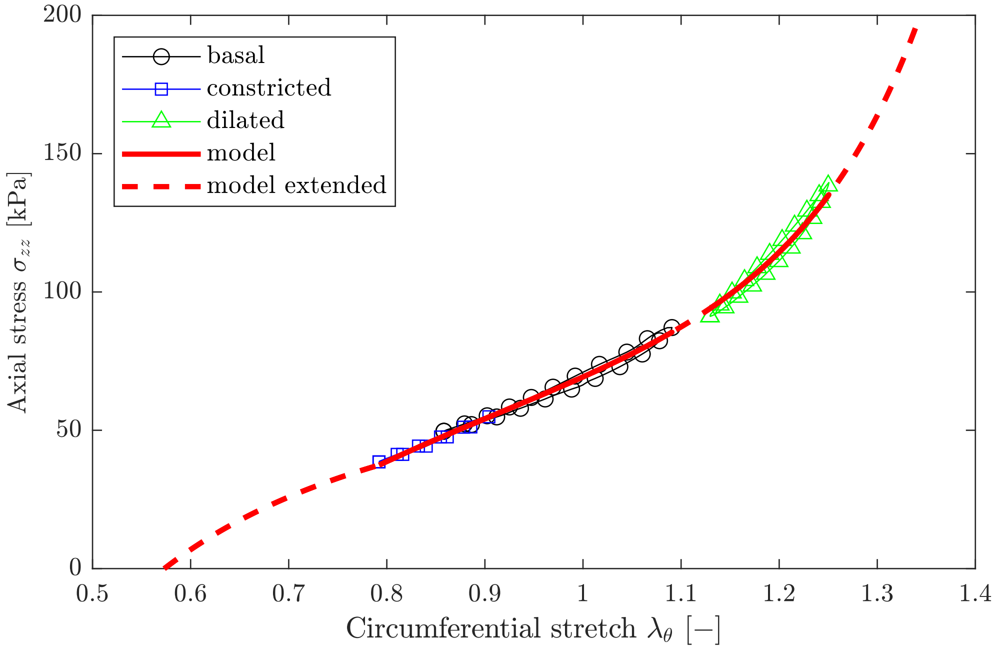

Fig. B3 Measured data and model prediction in terms of circumferential stress–circumferential stretch for subject II

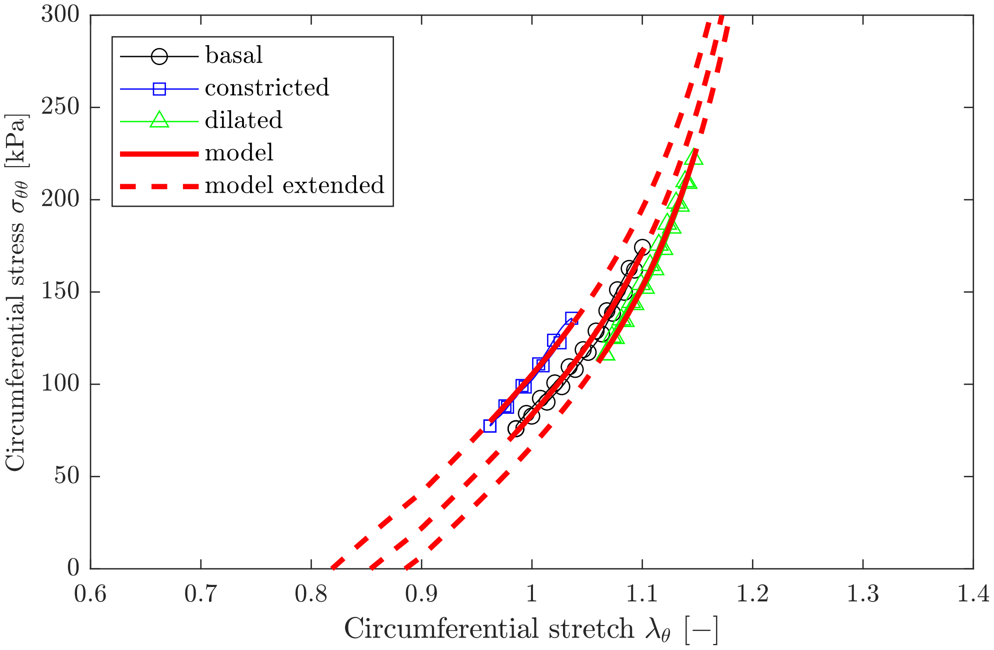

Fig. B4 Measured data and model prediction in terms of axial stress–circumferential stretch for subject II

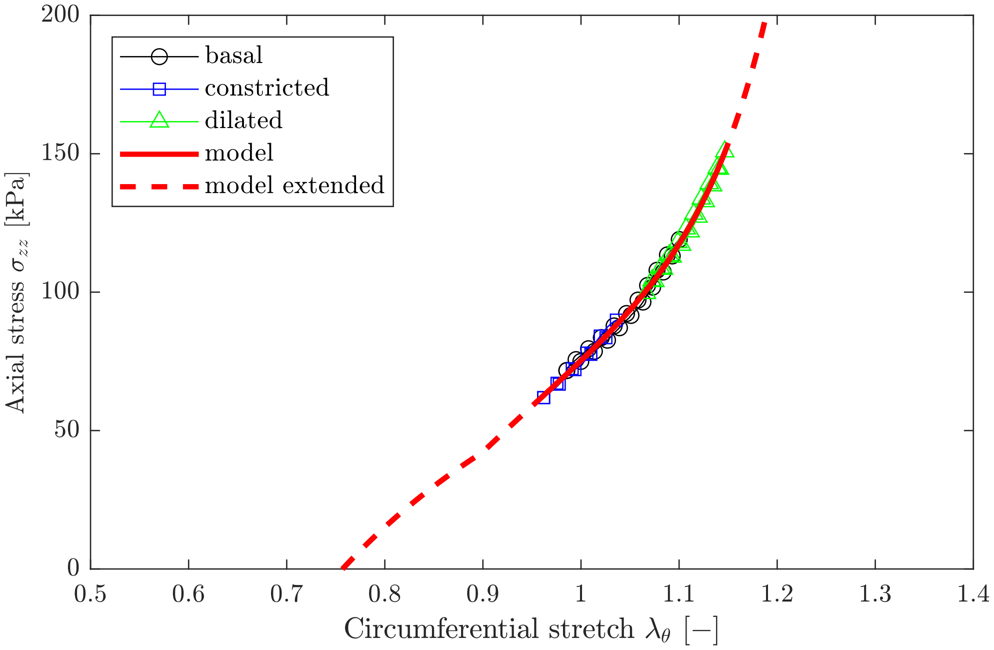

Supplement: Supplementary file 1 — Supplementary file1 (PDF 386 kb) [file 10237_2021_1462_MOESM1_ESM.pdf]
